# Identification of a candidate rice blast resistance gene, *Pior4*(t), in an introgression line of *Oryza rufipogon* using CRISPR/Cas9-mediated genome editing

**DOI:** 10.1270/jsbbs.24059

**Published:** 2025-03-26

**Authors:** Keiko Hayashi, Tomofumi Yoshida, Tarou Suzuki, Masaharu Kuroda, Yuriko Hayano-Saito

**Affiliations:** 1 Institute of Agrobiological Sciences, NARO, Kan-nondai, Tsukuba, Ibaraki 305-8604, Japan; 2 Mountainous Region Agricultural Institute, Aichi Agricultural Research Center, Inabu, Toyota, Aichi 441-2513, Japan; 3 Aichi Agricultural Research Center, Sagamine, Yazako, Nagakute, Aichi 480-1193, Japan

**Keywords:** *Oryza sativa*, *Pyricularia oryzae*, *Oryza rufipogon*, resistance gene, panicle blast, leaf blast

## Abstract

Resistance breeding for rice blast is an economic strategy for protecting rice crops against this disease. Genes with nucleotide-binding site leucine-rich repeat (NBS-LRR) structures are known to contribute to disease resistance. Here, we identified a candidate resistance gene, named *Pior4*(t), associated with leaf and panicle blasts in an introgression line carrying the chromosome 4 segment of wild rice (*Oryza rufipogon* Griff.) backcrossed with the cultivar ‘Nipponbare’ (*Oryza sativa* L.). Mapping analysis based on leaf blast severity confirmed that *Pior4*(t) was localized in the 177-kb NBS-LRR cluster region. To identify the *Pior4*(t) sequence, mutant lines were generated by knocking out a candidate NBS-LRR gene in a homozygous line carrying *Pior4*(t), M18, using CRISPR/Cas9-mediated genome editing. Leaf blast resistance was lost in the mutant lines lacking the corresponding Os04g0620950 N-terminal sequence of the M18 line. The result suggested that the counterpart NBS-LRR gene in the M18 line is involved in resistance to leaf blast. *Pior4*(t) showed homology to *Pi63* in the resistant cultivar ‘Kahei’, and an NBS-LRR gene in the resistant cultivar ‘Mine-haruka’ carrying *Pi39*(t). These results suggest that the NBS-LRR gene is a candidate gene of *Pior4*(t) and is present on the long arm of chromosome 4.

## Introduction

Rice blast, caused by the fungus *Pyricularia oryzae*, is one of the most destructive diseases affecting rice crops worldwide. Application of resistance (*R*) genes has emerged as an economical strategy in resistance breeding programs. Several studies have attempted to identify *R* genes against *P. oryzae* and introduce them into rice cultivars. Whole-genome sequencing has enabled the mapping of the most characterized *R* gene structure, the nucleotide-binding site-leucine-rich repeat (NBS-LRR) sequence (Zhou *et al.* 2004). Combining sequencing with transformation techniques has facilitated the identification of numerous functional NBS-LRR genes (Zhang *et al.* 2015).

NBS-LRR clusters have been identified in the long arm of chromosome 4 in the ‘Nipponbare’ genome (Zhou *et al.* 2004). Functional NBS-LRR genes against two rice diseases have been reported from this region: *Pi63* (Os04g0620950) and *OsPiPR1* (Os04g0621700) against rice blast and *Xa1* (Os04g0622600) against bacterial blight; the genes do not have an allelic relation (Liu *et al.* 2020, Xu *et al.* 2014, Yoshimura *et al.* 1998). Additionally, quantitative trait loci (QTLs) for rice blast have revealed many functional *R* genes in this region (around 29–33 cM, based on the ‘Nipponbare’ sequence) (Endo *et al.* 2012, Mizobuchi *et al.* 2014, Netpakdee *et al.* 2022, Terashima *et al.* 2008, Urso *et al.* 2016, Xu *et al.* 2008). A combination of three QTLs, designated *qBR4-2a*, *qBR4-2b*, and *qBR4-2c*, is involved in enhancing resistance in the resistant cultivar ‘Owarihatamochi’ (Fukuoka *et al.* 2012). The region containing *R* gene clusters is a useful target for introducing resistance to rice blast in cultivars through marker-assisted breeding.

Wild rice (*Oryza rufipogon* Griff.) could be a donor of *R* genes against rice blast. In introgression lines derived from *O. rufipogon* in the genetic background of *japonica* cultivar, a segment retaining partial resistance to rice blast has been identified on the long arm of chromosome 4 (Hirabayashi *et al.* 2010). Genome-wide re-sequencing of lines developed from *O. rufipogon* has revealed that many NBS-LRR clusters are present on chromosome 4 (Liu *et al.* 2017). Characterization of NBS-LRR genes derived from *O. rufipogon* accessions may provide genetic and phenotypic information on *R* genes on the long arm of chromosome 4.

In the present study, we identified the colocation of the resistance gene with the *R* gene cluster on the long arm of chromosome 4, named *Pior4*(t), using an F_2_ mapping population derived from an *O. rufipogon* W630 introgression line. The leaves and panicles of the line carrying the resistance region in the genetic background of ‘Nipponbare’ showed resistance to rice blast in the experimental field. Using CRISPR/Cas9-mediated genome editing, we attempted to identify whether resistance was due to an NBS-LRR gene in the NBS-LRR cluster region.

## Materials and Methods

### Plant materials and whole-genome sequencing

AsN145, an *O. rufipogon* W630 introgression line in the ‘Nipponbare’ background, was developed via two backcrosses to ‘Nipponbare’ and genome-wide screening with simple sequence repeat (SSR) markers; a wild accession of *O. rufipogon* W630, originating from Myanmar, was obtained from the National Institute of Genetics, Japan (Thanh *et al.* 2011). Resistance locus derived from any *O. rufipogon* W630 introgression line has not been used as a genetic resource in Japanese rice breeding systems previously. Line 8-3-10 was developed from a cross between AsN145 and ‘Nipponbare’. The F_2_ segregation population obtained by crossing 8-3-10 and ‘Nipponbare’ was genotyped, and F_3_ seedlings were used for phenotyping. The SSR markers used in the mapping experiments are listed in the Gramene Markers Database (https://www.gramene.org/markers/). PNK7 was used as the indel marker (Saito *et al.* 2010). Homozygous F_4_ lines M18, M29, M30, M57, M58, M64, M66, and M76 were developed from the F_3_ lines using SSR markers. The genotype at the *Pior4*(t) region in the F_4_ lines was confirmed by comparing the sequence amplified with primers to the sequences of ‘Nipponbare’ and 8-3-10 (Supplemental Table 1). The F_4_ lines of M29, M30, M57, M58, M64, M66, and M76 were used for mapping, and the M18 line was used for generating knocked out lines using CRISPR/Cas9-mediated genome editing.

Whole-genome sequencing of 8-3-10 and M18 was conducted using a BGISEQ-500 desktop sequencer, and single-nucleotide polymorphism (SNP) annotation was performed using a BGI system, according to the manufacturer’s instructions (BGI, Shenzhen, China). The genome sequence of ‘Nipponbare’ was used as a reference (Os-Nipponbare-Reference-IRGSP-1.0: https://rapdb.dna.affrc.go.jp/). The gap region in the whole-genome sequencing data of M18 genome sequence between S2-101 and S14-33 was estimated by zero coverage in a coverage plot, representing the number of reads per base pair, using the Integrative Genomics Viewer (IGV) application (https://igv.org).

### Assay of field resistance to leaf and panicle blast

Leaf and panicle blast severity in ‘Nipponbare’ and 8-3-10 was evaluated in an upland nursery field and an experimental high-disease-pressure paddy field at the Mountainous Region Agricultural Institute of the Aichi Agricultural Research Center (35°12.7ʹN, 137°30.4ʹE), respectively. In the field, the pathogenic Japanese race (007.0) is dominant (Ishihara *et al.* 2014). Leaf and panicle blast conditions were evaluated as previously described (Hayano-Saito *et al.* 2024).

Leaf blast severity in each line was evaluated using the disease severity index (Asaga 1981) in seedlings at 55 (2018) and 57 days (2019) of age. The scores ranged from 0 (no leaf symptoms) to 10 (damage to the entire plant).

Fifteen ‘Nipponbare’ and 8-3-10 plants were transplanted in 90-cm-long rows at 30-cm intervals. An average of 2–4 main panicles per plant and 50 (2017) and 40 (2018) samples from each row were used in the evaluation. We assessed panicle blast severity according to the location of damage to panicle tissues (Hayano-Saito *et al.* 2024). Panicles with white head symptoms caused by damage to the rachis node, rachis internode, and neck node were counted.

### Plant growth conditions and inoculation with leaf blast

The F_3_ and F_4_ plants were grown in a greenhouse, inoculated artificially by spraying with *P. oryzae* isolates (Ao92-06-2: race 337.1), and evaluated using the disease severity index as described previously (Hayashi *et al.* 2016). To quantify the percentage of diseased leaf area in the homozygous F_4_ lines, we used digital imaging (Hayashi *et al.* 2019). Digital images of infected leaves at 7 days post-inoculation stained with blue cut-flower dye (Flower Fantasy; Palace Chemical, Yokohama, Japan) were captured from 10 plants (Supplemental Fig. 1). The 10 dyed leaf samples were pasted on white-colored tape, and images were captured using a digital camera (Tough TG-5; Olympus) and then processed using a digital microscope software (VHX-5000; Keyence) to extract regions corresponding to leaf tissues damaged by hue (leaf, 47–75), saturation (leaf, 13–108), and brightness (leaf, 128–255). Whole leaf regions of the 10 leaves were extracted by hue (leaf, 32–156) and saturation (leaf, 21–255). The extracted areas were quantified using a digital microscope software (Supplemental Fig. 1).

Diseased leaf area (%) = (Damaged leaf area)/(Whole leaf area) × 100.

### Generation of CRISPR/Cas9-edited mutant rice plants

To introduce mutations into the rice genome, we used the binary vector pZNH2GTRU6 (Manuscript in preparation). This vector is a derivative of the pZ2028 plasmid (Kuroda *et al.* 2010), which contains hygromycin phosphotransferase driven by the nopaline synthase promoter, *Streptococcus pyogenes* Cas9 driven by the modified rice polyubiquitin promoter, and four guide RNAs for editing target genes driven by the rice U6-2 non-coding RNA promoter. The unique sequence of the 19-bp targeted sequence for guide RNA was selected manually and checked using a Basic Local Alignment Search Tool (BLAST) in the Rice Annotation Project Database (RAP-DB). *Agrobacterium*-mediated transformation procedure has been described previously (Hayashi and Yoshida 2009, Kuroda *et al.* 2010).

T_0_ plants were genotyped through PCR-amplified patterns using the LRRf1 and LRRr1 primer pairs to select mutant lines showing a deletion at the target site on 2.5% agarose gel (Supplemental Table 2). T_1_ and T_2_ plants were genotyped via PCR-amplified patterns using the LRRf1 and LRRr1 primer pairs to screen for homozygous plants. T_1_ and T_2_ plants, used for phenotyping, were grown until the 5.5-leaf stage in a growth chamber (BioTRON LH-240SP; NK System, Tokyo, Japan) under 15-h light (23°C for 1 h, 26°C for 13 h, and 23°C for 1 h) and 9-h dark (20°C) conditions and used for artificial inoculation by spraying the *P. oryzae* isolates (Ao92-06-2). Susceptible lesions in the 4th to 6th leaf stages were counted at 10 (T_1_) and 7 days (T_2_) post-inoculation, respectively.

### cDNA cloning and expression analysis

Total cellular RNA was extracted using the TRIzol method (Thermo Fisher Scientific K.K., Tokyo, Japan). RNA samples (2 μg) were converted to cDNA using SuperScript III (Thermo Fisher Scientific K.K.) and used for cDNA cloning and reverse transcription-polymerase chain reaction (RT-PCR). The 5ʹ-ends of cDNA clones were identified using a 5ʹ-full RACE Core Set (Takara, Shiga, Japan). The 3ʹ-end of cDNA was deduced using several primers developed by the common sequence between ‘Nipponbare’ sequence and whole-genome sequencing data of M18. Expression analysis of *Pior4*(t) was performed using Pi_K_f4 (5ʹ-UTR position) and Pi_r5 primer (3ʹ-UTR position) pairs, which were designed to amplify full-length cDNA. Rice glyceraldehyde 3-phosphate dehydrogenase (*OsGAPDH*) was used as the reference gene (Hayashi *et al.* 2022). The editing positions of cDNA was checked using sequencing and genotyping was performed using the LRRf1 and LRRr1cDNA primer pairs. The primers used are listed in Supplemental Table 2.

### Sequence homology analysis

Sequence homology analysis was performed using the GENETYX software (Ver 16; GENETYX Corp., Tokyo, Japan). The NB-ARC and LRR domains were predicted using the InterPro database (Paysan-Lafosse *et al.* 2023). The nucleotide sequences generated in this study have been deposited in the DNA Databank of Japan (DDBJ) (Accession numbers: LC833302 and LC833303).

### Statistical analysis

Statistical analyses were performed using the Bell Curve in Excel developed by the Social Survey Research Information Co., Ltd.

## Results

### Field resistance to leaf and panicle blast in the *O. rufipogon* W630 introgression line

Line 8-3-10, which was an *O. rufipogon* W630 introgression line in the ‘Nipponbare’ background, showed resistance to leaf and panicle blast under the field conditions for 2 years (Tables 1, 2). Whole-genome re-sequencing confirmed that 8-3-10 retained a *O. rufipogon* W630 chromosome segment of 29.338 Mb (based on the complete sequence of ‘Nipponbare’ AP014960.1) on the long arm of chromosome 4 and had no significant introgressed fragments on the other chromosomes (Supplemental Fig. 2A). These findings suggest that the resistance gene responsible for leaf and panicle blast resistance was located on this chromosome segment.

### Mapping of *R* gene in the *O. rufipogon* W630 introgression line

**Fig. 1.**
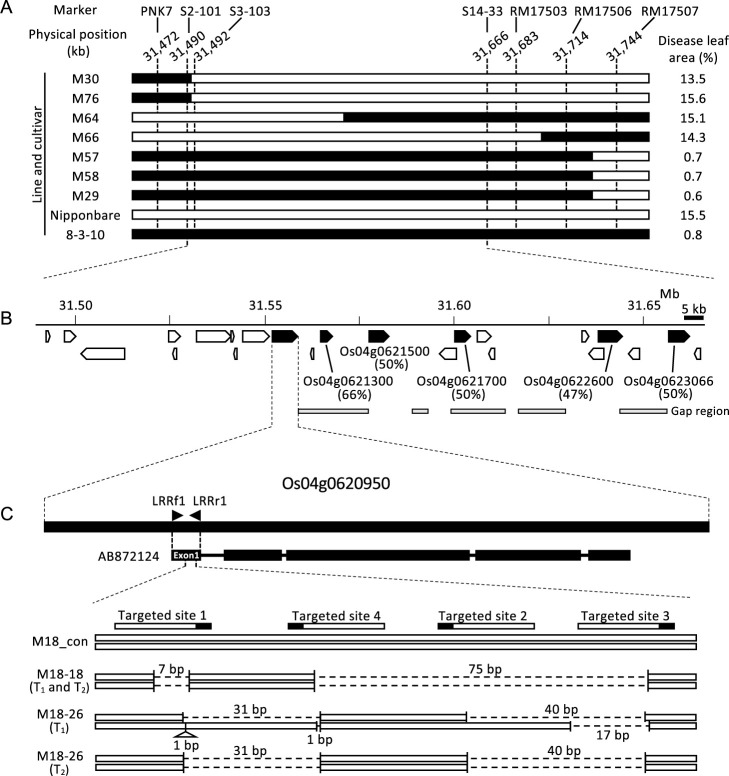
Physical map of *Pior4*(t). A, Mapping. Black bars indicate the region derived from the *O. rufipogon* W630 introgression line and white bars indicate the region derived from the cultivar ‘Nipponbare’. Marker positions were estimated from the ‘Nipponbare’ sequence (IRGSP-1.0). B, Gene position in ‘Nipponbare’. Pentagon represents the open reading frames (ORFs) predicted using RAP-DB. ORFs predicted as NBS-LRR structures are marked in black. The max identity between Os04g0620950 and the five predicted NBS-LRR structures is shown in parentheses. The gap regions in the whole-genome sequencing data of the M18 line are represented with gray bars under the ORFs. C, Schematic map of the target sites on a *Pior4*(t) candidate gene and the sequence of T_1_ and T_2_ mutant lines. The upper part shows the positional relation between Os04g0620950 and *Pikahei-1*(t) mRNA (AB872124). The ORF information of AB872124 was based on the genomic sequence (AB872116). Four target sites were designed in the N-terminal region of Os04g0620950 for CRISPR/Cas9-mediated genome editing. LRRf1 and LRRr1 primers used to screen T_0_ plants are shown. The sequencing results of M18_con (transformed with the binary vector pZNH2GTRU6) and T_1_ and T_2_ mutant lines are shown schematically. Open boxes represent wild-type M18 sequences and dotted lines represent deletions. The complete sequence is shown in Supplemental Fig. 3A.

To map the resistance gene, we assessed 1,293 F_2_ segregating plants from the cross between 8-3-10 and ‘Nipponbare’ (Fig. 1A). Seven F_2_ lines were selected using PNK7 and RM17507, and the delimitation region was 272 kb. Homozygous lines were developed from each line and disease severity was assessed. The percentage of the diseased leaf area in four lines (M30, M76, M64, and M66) was the same as that for ‘Nipponbare’, and the percentage of the diseased leaf area in other three lines (M57, M58, and M29) was the same as that for 8-3-10 (Fig. 1A). The results indicated that the resistance gene was localized in a 177-kb region based on the ‘Nipponbare’ genome sequence between S2-101 and S14-33 (Fig. 1B). Additionally, 23 open reading frames (ORFs) were predicted in the Rice Annotation Project Database (RAP-DB); among them, six ORFs showed NBS-LRR sequence-related structures (Os04g0620950, Os04g0621300, Os04g0621500, Os04g0621700, Os04g0622600 and Os04g0623066) (Kawahara *et al.* 2013, Sakai *et al.* 2013). The region containing these ORFs is colocalized with the rice blast resistance genes *qBR4-2b*, *PiPR1*, and *Pi63* (*Pikahei-1*(t)) (Fukuoka *et al.* 2012, Liu *et al.* 2020, Xu *et al.* 2014). Thus, we hypothesized that an *R* gene is present in the *R* gene cluster in 8-3-10, and it was temporarily named *Pior4*(t) (or4 stands for *Oryza rufipogon* chromosome 4).

### Identification and characterization of *R* gene in the *O. rufipogon* W630 introgression line

To identify the *R* gene structure located in the 177-kb region, we used the M18 line instead of the F_4_ lines shown in Fig. 1A, because M18 retained the minimal region derived from 8-3-10 in our developed homozygous F_4_ lines. Whole-genome sequencing results revealed that the M18 line retained a 1.3-Mb *O. rufipogon* W630 chromosomal segment, including the region between PNK7 and RM17507 (Fig. 1A, Supplemental Fig. 2B). Additionally, several gap regions were identified in the region predicted to contain NBS-LRR sequence-related structures (Fig. 1B). The results indicated that the genomic structure of M18 was different from that of ‘Nipponbare’, and the sequencing data of M18 was of low quality for further analyses. Thus, we focused on the sequence of the rice blast resistance gene *Pi63* (*Pikahei-1*(t)) in this study (Xu *et al.* 2014).

The first exon of *Pikahei-1*(t) mRNA (GenBank accession number: AB872124) showed 96% identity with the first estimated exon of Os04g0620950 (Fig. 1C). Additionally, the whole-genome sequencing data of M18 were available for this region. Thus, we hypothesized that the corresponding Os04g0620950 in the M18 line is a candidate of *Pior4*(t) and knocked out the gene in the M18 line using CRISPR/Cas9-mediated genome editing.

We designed four targeted sites in the first estimated exon of Os04g0620950 whose sequence was identical to that of ‘Nipponbare’ and M18 (Fig. 1C, Supplemental Fig. 3A, 3B). Genomic sequence of Os04g0620950 showed 47%–66% maximal identity with that of Os04g0621500, Os04g0621700, Os04g0622600, Os04g0623066, and Os04g0621300 (Fig. 1B); however, in the case of Os04g0621300, the sequence only coded for LRR domains. The 136-bp targeted sequence in the first estimated exon of Os04g0620950 showed 62%–66% maximal identify with that of Os04g0621500 (62%), Os04g0621700 (66%), Os04g0622600 (65%), and Os04g0623066 (64%) (Supplemental Fig. 3B). The sequences of the four guide RNAs did not completely align with those sequences in the ‘Nipponbare’ genome.

In the generated 30 T_0_ plants, genotyping using the LRRf1 and LRRr1 primer pairs showed that 18 plants exhibited the deletion-band pattern on 2.5% agarose gel: 13 plants exhibited multi-band pattern, whereas 5 plants exhibited single-band pattern (Supplemental Table 3). Among these 5 plants, 2 had over 50-bp deletion (M18-18 and M18-26) and were used for homozygous selection. We deduced that a large deletion is a rare event at the target site; even if gene duplication has occurred in the cluster of M18 line, over 50-bp deletion would not occur in the duplicated gene as well as the editing-targeted gene within one T_0_ line. Therefore, we screened the T_1_ and T_2_ plants with a large deletion specifically at the first estimated exon of the corresponding Os04g0620950 gene on agarose gel.

Two T_1_ lines of M18-18 and M18-26 were found to have several deletions/insertions at the target site (Fig. 1C, Supplemental Fig. 3A). An artificial inoculation assay using *P. oryzae* isolates (Ao92-06-2) revealed that compared with the control M18 (M18_con), M18-18 and M18-26 lost their resistance function (Table 3). Similar inoculation results were observed in the homozygous T_2_ lines M18-18 and M18-26 (Table 3). These two lines showed no significant phenotypic difference except for blast resistance from M18_con.

**Fig. 2.**
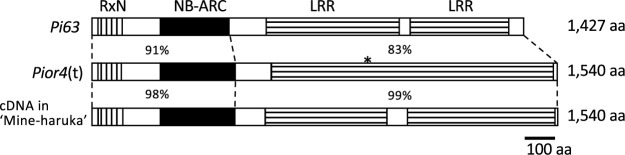
Deduced amino acid sequence identity of *Pior4*(t), *Pi63* (GenBank accession number: BAO79825), and the cDNA identified in ‘Mine-haruka’. The RxN, NB-ARC, and LRR domains predicted using the InterPro database are shown schematically. Amino acid sequence of ‘Nipponbare’ exhibited high homology with *Pior4*(t) (98%: 1–902 aa sequence), but a nonsense mutation was caused by the stop codon (at codon 903; the position of stop codon is marked with an asterisk).

Next, based on the sequences of Os04g0620950 and *Pikahei-1*(t) mRNA (Gene Bank accession no. AB872124) and the corresponding sequence data of M18, we isolated and identified the cDNA sequence of the corresponding Os04g0620950 gene in M18. We also confirmed that the sequence of cDNA clones derived from M18-18 and M18-26 showed the same deletion/insertion patterns as the edited genomic sequences. The expression of the edited-corresponding Os04g0620950 gene in M18-18 and M18-26 was at the same level as the non-edited corresponding Os04g0620950 gene in M18_con (Supplemental Fig. 4). These data suggested that the isolated cDNA cording with the NBS-LRR structure was a candidate gene of *Pior4*(t) that was targeted by CRISPR/Cas9-mediated genome editing. In the comparison of the coding sequences of *Pior4*(t) and *Pi63*, *Pior4*(t) exhibited homology with 91% N-terminal domain (RxN and NB-ARC) and 83% LRR domain of *Pi63*, respectively (Fig. 2).

We identified the coding sequence of cDNA in the resistant cultivar ‘Mine-haruka’ carrying *Pi39*(t) based on the information of *Pior4*(t)-type sequence. *Pi39*(t) is located close to RM3843 (the marker position based on ‘Nipponbare’ sequence: 31,683 kb) in Chubu 111 (Terashima *et al.* 2008). This gene is involved in leaf and panicle blast resistance (Hayano-Saito *et al.* 2024) and has been used as a genetic resource in Japanese rice breeding systems. Consequently, we isolated cDNA from ‘Mine-haruka’, and identified the sequences. The cDNA sequence showed homology to *Pi63* and *Pior4*(t) (Fig. 2). This result implied that the cDNA clone can be used as a candidate gene in future studies aiming to identify *Pi39*(t).

## Discussion

Resistance breeding research for rice blast has shown that resistance to leaf blast generally correlates with resistance to panicle, although there are a few exceptions (Bonman 1992, Hayano-Saito *et al.* 2024, Hayashi *et al.* 2010, Ishihara *et al.* 2014, Ou 1985). In the present study, we confirmed resistance gene properties in an experimental field. Leaf blast resistance was observed in 8-3-10. However, in the panicles, white head symptoms due to damage to the rachis and neck node were observed, but the level of damage was lower in 8-3-10 than in ‘Nipponbare’. The results suggest that lines carrying *Pior4*(t) exhibited leaf and panicle resistance, resulting from the relatively strong resistance of leaves in the plants grown in the experimental field (Tables 1, 2). Pathogenic race (007.0), which is dominant in an experimental field, is the most prevalent strain in Japan (Ishihara *et al.* 2014, Koizumi *et al.* 2007), indicating that *Pior4*(t) is a potential gene resource for breeding rice blast-resistant varieties in Japan.

The *R* genes have generally been identified using QTL analysis in greenhouse and experimental field populations (Ballini *et al.* 2008). However, the sample numbers for gene identification were limited in this study owing to time and space constraints. Our mapping population showed that an NBS-LRR cluster was localized in a 177-kb region but did not reveal the *Pior4*(t) structure within the gene cluster. Functional NBS-LRR genes for two diseases have been reported previously in the region from the same region: *Pi63* and *OsPiPR1* for rice blast and *Xa1* for bacterial blight, all of which are non-allelic genes (Liu *et al.* 2020, Xu *et al.* 2014, Yoshimura *et al.* 1998). The sequences of the NBS domains are highly conserved and are used as data for phylogenetic analyses of NBS-LRR-encoding genes (Zhou *et al.* 2004). Thus, we deduced that the N-terminal region (side of the NBS region) is a good target for gene editing compared with the C-terminal region (side of the LRR region) and created knockout mutants by targeting the *Pi63*-type sequence using CRISPR/Cas9-mediated genome editing (Fig. 1C, Supplemental Fig. 3A). Homozygous-deletion mutants lacking the RxN sequence of the counterpart gene of Os04g0620950 in the M18-18 and M18-26 lines lost their resistance (Table 3, Fig. 2). There is a slight possibility that homologous NBS sequences of Os04g0620950 located on the other chromosome segments affected the resistance, as M18 was developed as a homozygous line retaining the 1.3-Mb resistant chromosome segment in the background of a susceptible ‘Nipponbare’ chromosome (Supplemental Fig. 2B). However, our study did not confirm whether the other predicted genes in the 177-kb region of the M18-18 and M18-26 lines have off-target mutations, especially mutations of small deletions and/or insertions, because complete whole-genome sequencing data of the M18-18 and M18-26 lines were not analyzed. Although the possibility of off-target effects in the resistance-related genes in the 177-kb region is difficult to eliminate, the results suggest that the candidate gene of *Pior4*(t) is the counterpart sequence of Os04g0620950 in M18.

Many rice blast-resistance genes have been reported in and around this region in both *japonica* and *indica* subspecies (Fukuoka *et al.* 2012, Mizobuchi *et al.* 2014, Terashima *et al.* 2008, Urso *et al.* 2016, Xu *et al.* 2008, Zhang *et al.* 2015). In the present study, the RxN and NB-ARC sequences of *Pior4*(t) were highly homologous to those of *japonica* ‘Nipponbare’ and *japonica* upland cultivar ‘Kahei’. Additionally, isolated cDNA clone from a *japonica* cultivar, ‘Mine-haruka’, in which *Pi39*(t) is derived from the Yunnan upland cultivar ‘Haonaihuan’ (Terashima *et al.* 2008), showed high homology to *Pior4*(t) (Fig. 2), although the function of the gene in ‘Mine-haruka’ has not been confirmed yet. Thus, the candidate gene of *Pior4*(t) might have homologous genes in a wide range of cultivars.

Pyramiding and multiline breeding are attractive strategies to enhance disease resistance; however, detection of various resistant genotypes during the breeding process is essential. A rapid SNP genotyping system was developed in Japan to detect 10 race-specific blast-resistance loci for breeding programs (Kitazawa *et al.* 2019). Allele sequence information of *Pior4*(t) can contribute to the development of such an *R* gene genotyping system including traditional DNA markers and to advances in genetic studies and breeding strategies. The technique of identifying a candidate *R* gene structure using CRISPR/Cas9-mediated genome editing can be applied to a wide array of crops for future disease-resistant breeding programs.

## Author Contribution Statement

T.Y. and T.S. maintained the experimental paddy field and assessed leaf blast in the field. Y.H-S. conducted panicle blast evaluation. M.K. performed CRISPR/Cas9-mediated genome editing. K.H. conducted all experiments and wrote the manuscript. All authors have read and approved the final version of the manuscript.

## Supplementary Material

Supplemental Figures

Supplemental Tables

## Figures and Tables

**Table 1.** Leaf blast severity score of the 8-3-10 line grown in the experimental field

Cultivar/line	2018					2019			
	Rep. 1	Rep. 2	Rep. 3	Average		Rep. 1	Rep. 2	Rep. 3	Average
Nipponbare	5.0	4.0	6.0	5.0		5.0	5.0	5.0	5.0
8-3-10	1.0	0.0	0.5	0.5		0.5	0.5	0.5	0.5

**Table 2.** Number of panicles with damaged rachis and/or neck node in 8-3-10 and ‘Nipponbare’ in the experimental field

	2017					2018			
	Investigation days after heading	Total number	Number of panicles with scores 5–7* (%)			Investigation days after heading	Total number	Number of panicles with scores 5–7* (%)	
			Nipponbare	8-3-10				Nipponbare	8-3-10
Rep. 1	37–38	50	46 (92.0)	13 (26.0)		20	40	9 (22.5)	4 (10.0)
Rep. 2	38	50	43 (86.0)	20 (40.0)		18–19	40	23 (57.5)	3 (7.5)
Rep. 3	42	50	44 (88.0)	14 (28.0)		17–20	40	18 (45.0)	0 (0.0)

* Panicles with white head symptoms caused by damage to the rachis node (score 5), rachis internode (score 6), and neck node (score 7) were counted.

**Table 3.** Leaf blast resistance in CRISPR/Cas9-edited rice mutant plants

Generation	Line name	Number of susceptible lesions in each plant					Average	±SE
		1	2	3	4	5		
T_1_	M18_con	0	0				—	—
T_1_	M18-18	7	6				—	—
T_1_	M18-26	7*	7*				—	—
T_2_	M18_wt	3	3	4	5	6	4.2	±0.6 b
T_2_	M18_con	1	0	0	2	1	0.8	±0.4 b
T_2_	M18-18	28	35	37	36	23	31.8	±2.7 a
T_2_	M18-26	29	20	47	16	29	28.2	±5.3 a

M18_wt is an intact M18 line. M18_con is a line transformed using the binary vector pZNH2GTRU6. Significant differences (*p* < 0.01) are indicated with different letters based on Tukey’s test. * Heterozygous genotypes are shown using the LRRf1 and LRRr1 primers.
