# Supplementary material for: Identification of a candidate rice blast resistance gene, Pior4(t), in an introgression line of Oryza rufipogon using CRISPR/Cas9-mediated genome editing
Source: Breed Sci. 2025 Mar 26;75(2):139–46. doi: 10.1270/jsbbs.24059 (PMC12395198; doi:10.1270/jsbbs.24059)
Supplement: Supplementary file 1 — Supplemental Figures [file 75_139_s1.pdf]

Hayashi et al./Breeding Science
doi: 10.1270/jsbbs.24059

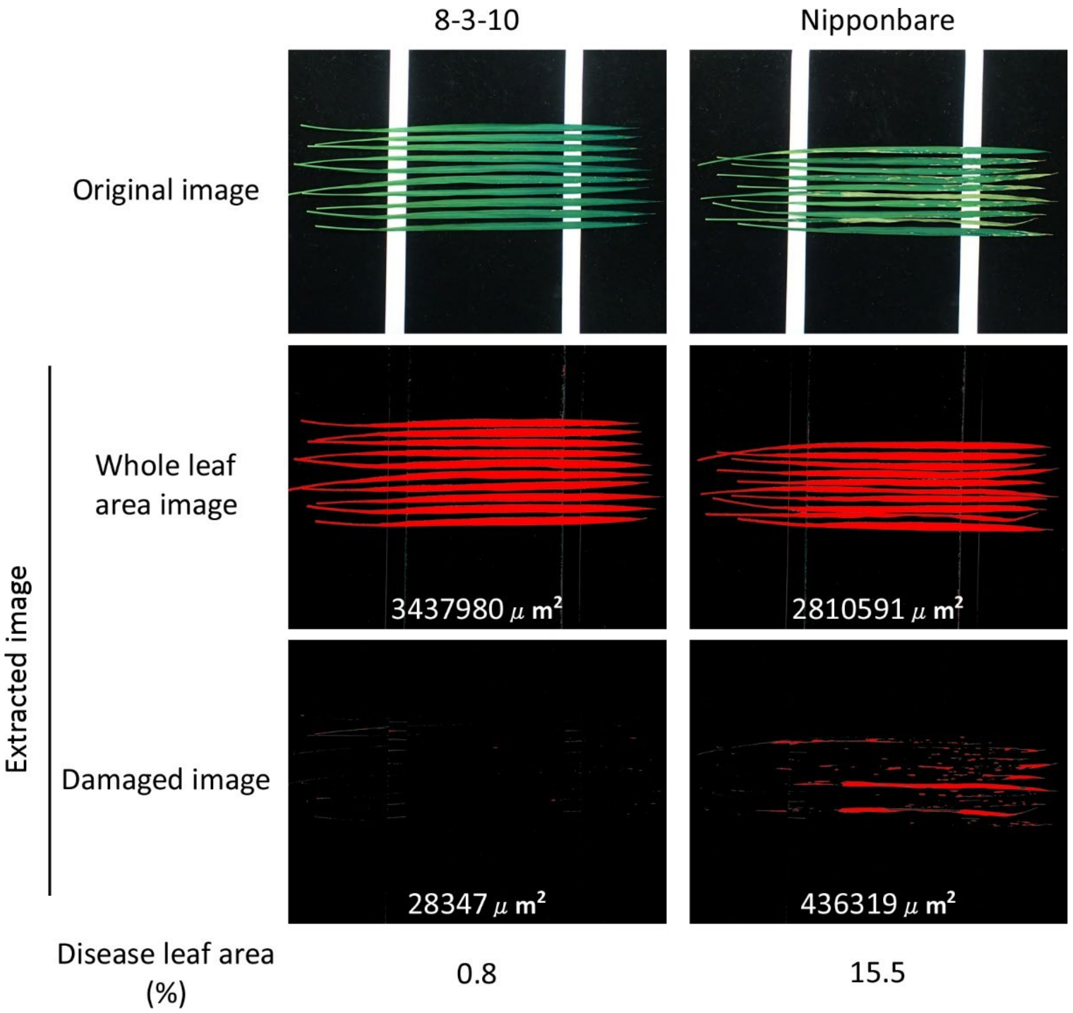


**Supplemental Fig. 1** Image-based phenotyping using cut-flower dye solution
Estimation of percentage of diseased leaf area using digital imaging. Representative images of 8-3-10 and 'Nipponbare'. Digital imaging of 10 leaves. The samples were stained with blue cut flower dye (upper). The red-on-black images, extracted using the Keyence microscopy software ("Extracted image"), were analyzed using the number of red areas on the image. The upper image shows the entire leaf area, and the lower image shows the damaged leaf area.

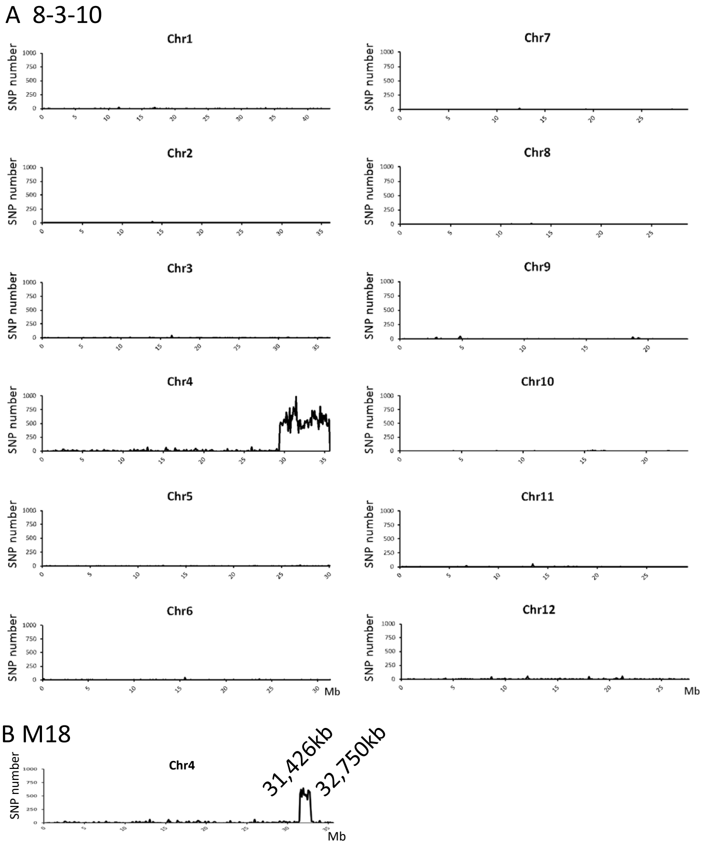


**Supplemental Fig. 2** Distribution of homotype SNPs in the 8-3-10 and M18 lines

The distribution of homotype SNPs per 100 kbp is shown for chromosomes 1–12 (A, 8-3-10) and 4 (B, M18). Predicted recombination positions in M18 are also shown. The x-axis represents the physical position (Mb). The published genome sequence of 'Nipponbare' was used as the reference sequence.

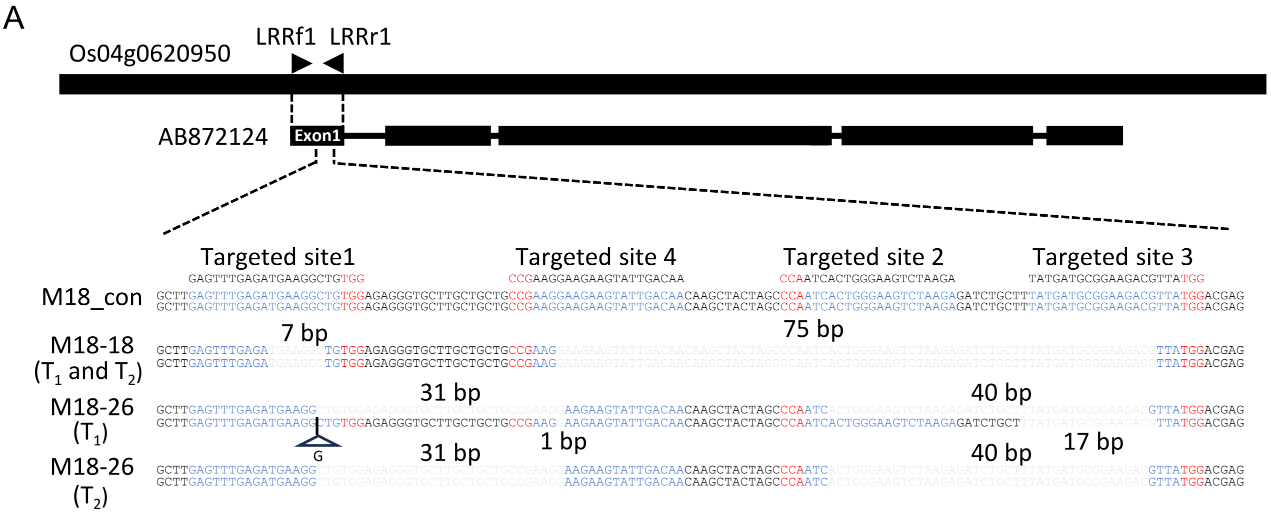


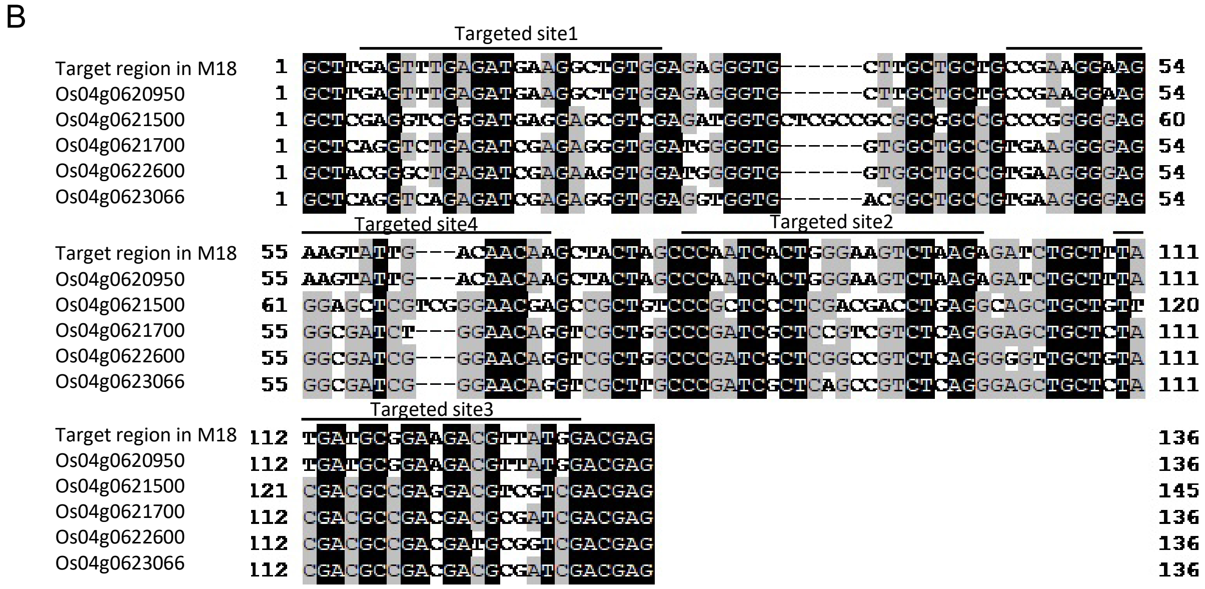


**Supplemental Fig. 3** Detailed sequence information of Fig. 1C

A, The target sequence (in blue) containing the PAM site (in red) is shown. The deleted sequence is shown in gray. Target sites 2 and 4 were targeted on the complementary strand. B, The relation between the sequence of four guide RNAs and the sequence of Os04g0621500, Os04g0621700, Os04g0622600, and Os04g0623066.

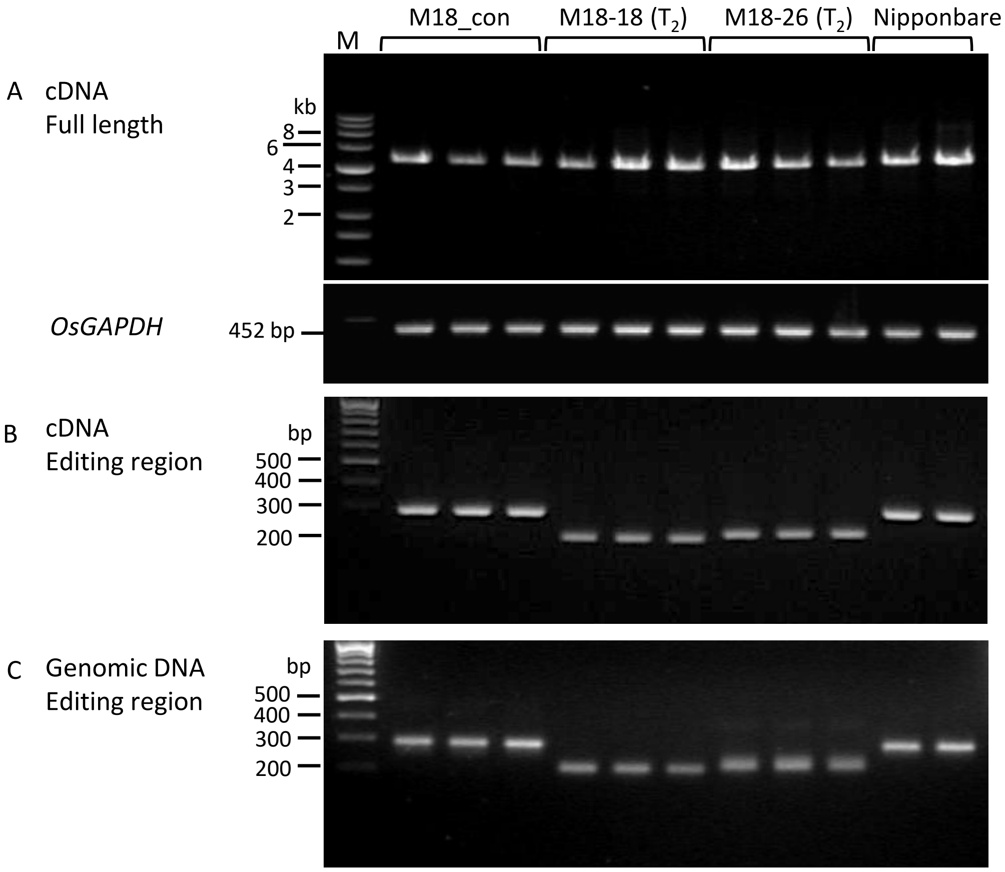


**Supplemental Fig. 4** Expressions and genotypes of *Pior*4(t)-allelic genes in M18, M18-18, M18-26, and 'Nipponbare'

A, Full length cDNA on 0.8% agarose gel. *OsGAPDH* was used as an expression-reference gene (452-bp long) on 2.0% agarose gel. B, Editing region of cDNA on 2.5% agarose gel. The amplified full-length cDNA products (A) were used as a PCR template. C, Editing region of genomic DNA on 2.5% agarose gel. M, Size marker of 0.5–12-kb perfect DNA marker (panel A, Novegen, Darmstadt, Germany) and 100-bp Gene Ladder (panel B-D, Nippon Gene Co., Ltd.).
