# Supplementary material for: Identification of a candidate rice blast resistance gene, Pior4(t), in an introgression line of Oryza rufipogon using CRISPR/Cas9-mediated genome editing
Source: Breed Sci. 2025 Mar 26;75(2):139–46. doi: 10.1270/jsbbs.24059 (PMC12395198; doi:10.1270/jsbbs.24059)
Supplement: Supplementary file 2 — Supplemental Tables [file 75_139_s2.pdf]

doi: 10.1270/jsbbs.24059

Supplemental Table 1. Markers used for mapping *Pior4*(t)

Marker	Type	Forward (5'-3')	Reverse (5'-3')
S2_101	Sequence	GCTGATTAGCTGAGTCCTACTG	CTGCAACAATGTGCTACTACCAGC
S3_103	Sequence	GACTCACGACGCATGATCTAATC	AGCAAACCGCTACTACAATCTC
S14_33	Sequence	GCATGTGCCAATGCTCCCGTG	GTAGCGCACGCTTGACGCTG

Supplemental Table 2. Sequence of primers used in the experiment

Primer	Forward (5′-3′)	Purpose
LRRf1	TGTGGTGGATGCTGCGATAG	T_0 screening<br>Genotyping the edited region in genomic DNA
LRRr1	TCATGAACCTTTTTCGATCC	
LRRr3_PHO	GCAGCACTGGAGCCTTCAC	5'-RACE
Pi_S2	CGACTACCACCGGCTCAAAC	
Pi_S1	TATCGAGAAGCTTGAGTTTG	
Pi_A1	GAGTCCAGGCTTCCATCTG	
Pi_A2	CAGTGAAGAAGCTCCCAAG	
Pi_K_f4	CCTTGGTGTGGTGAGAAATG	Full-length cDNA cloning and RT-PCR
Pi_r5	GGAAGTGATTGCAGAGCAGATAGC	
LRRf1	TGTGGTGGATGCTGCGATAG	Genotyping the edited region in cDNA
LRRr1cDNA	GCCTTCACCTTTTTCGATCC	
GAPDH_f	ACAACTGTTCATGCCATCAC	RT-PCR targeted to *OsGAPDH*
GAPDH_r	TCGATGACACGGTTGCTGTA	

Supplemental Table 3. Analysis number of the T_0 lines and their PCR genotyping patterns using LRRf1 and LRRr1 detected on 2.5% agarose gel.

Amplification pattern	Number of T_0 line
Same pattern as Nipponbare	11
Multi-band pattern	13
Single-band pattern (<50-bp deletion)	3
Singl-band pattern (>50-bp deletion)	2
No amplification	1
Total	30
